# Ovarian Crohn's Disease: A Case Report and Review of the Literature

**DOI:** 10.1155/2020/1826469

**Published:** 2020-10-06

**Authors:** Hamza Mohammed, Rana Bokhary, Mohammed Nassif, Mahmoud Mosli

**Affiliations:** ^1^Departments of Medicine, King Abdulaziz University, Jeddah, Saudi Arabia; ^2^Departments of Pathology, King Abdulaziz University, Jeddah, Saudi Arabia; ^3^Departments of Surgery, King Abdulaziz University, Jeddah, Saudi Arabia

## Abstract

We present to you a rare case of Crohn's disease involving the ovary in a 28-year-old nulligravida Eritrean patient. This is considered a rare manifestation of Crohn's disease that is believed to be due to fistulization between the ovary and intestines.

## 1. Introduction

Crohn's disease (CD) is an inflammatory bowel disorder (IBD) that is progressive in nature, resulting in certain complications such as fistulization or stricture formation that once developed may require surgical intervention [1]. The clinical picture of CD includes gastrointestinal symptoms such as abdominal pain, weight loss, and diarrhea [2], in addition to systemic manifestations such as joint pain and skin involvement [3, 4]. The age group for CD varies from childhood to older age groups, but it is most commonly diagnosed between 15 and 30 years of age [4]. The prevalence of CD worldwide and in Saudi Arabia has been increasing over the past 30 years. The most commonly affected site in CD is the ileo-cecal region [5].

There are no firm diagnostic features for CD but typical signs include perianal fistulas, large skin tags or abscesses on physical examination [6], small bowel involvement, mucosal ulceration, cobble stoning, narrowing, obstruction, and fistulas seen on cross-sectional imaging [6], as well as ileo-cecal valve stenosis, cobble stoning, ulcerations, strictures, and fistulas seen endoscopically [6]. Histopathological features suggestive of CD include noncaseating granulomas and transmural lymphoid aggregates, which are often only noticeable after examining a resected part of the bowel [6]. Differentiating CD from intestinal tuberculosis remains a diagnostic challenge and often results in a delay in the diagnosis and treatment [7, 8]. Both CD and intestinal TB can result in fistulization into adjacent organs, commonly into other small or large bowel segments, the urinary bladder, the vagina, or even to the skin. There are also reports of fistulization into the stomach and duodenum [9–11]. Here, we present a case of a young female presenting with a rare complication of CD, which involved the ovary.

## 2. Case Presentation

A 28-year-old single nulligravida Eritrean patient presented in 2013 to the emergency department with right lower quadrant pain for 3 weeks. Her pain started gradually; it was colicky and throbbing in nature and radiated to her right leg. The pain was aggravated by fatty and spicy food, occurred on and off, and was partially alleviated by painkillers. The pain was associated with nausea, vomiting, bloating, alternating diarrhea and constipation, and episodes of bleeding per rectum. The patient had her first menstrual period when she was 11; she had regular cycles for 7 days until recently, as her period lasted only for 3 days in the two cycles preceding her presentation, with increased premenstruation pain. She reported that she had no weight loss, no loss of appetite, no night sweats, but had palpitations while her other systems were unremarkable. She only complained of eczema and her past surgical history was of an anal fistula in 2010; she had no blood transfusion and no known food or drug allergies, and she is a nonsmoker. Her younger sister was known to have ileal Crohn's disease.

She was diagnosed in the ER with appendicitis and underwent emergency appendectomy. After appendectomy, she continued to complain of continuous abdominal discomfort. In March of 2017, the patient returned to the hospital due to recurrence of pain associated with loss of appetite. A CT scan of the abdomen revealed a right adnexal mass inseparable from a thickened and stenosed distal ileum. An exploratory lower midline laparotomy was done that revealed an abscess in the right side of the abdomen in the vicinity of the distal part of the small intestines along with ovarian swelling. She underwent abscess drainage and right ovarian cystectomy only, as the ileum itself was inflamed but with no fat creeping and was not fibrotic, indicating that it itself was not diseased but showed signs of inflammation due to proximity to the diseased ovary. The patient tolerated the procedure well, had an uneventful hospital stay, and was discharged from the hospital three days later.

**Figure 1:**
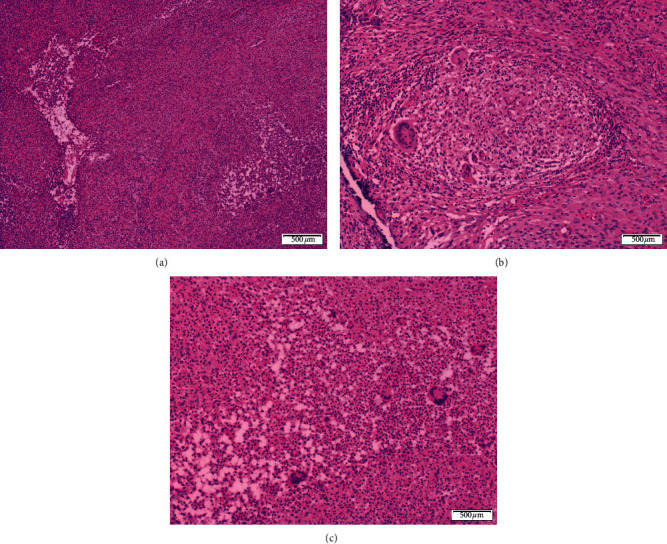
Histopathological findings in the resected ovarian mass. (a) Low-power view showing diffuse mixed inflammatory infiltrate of the ovarian stroma with the formation of a microabscess “on the left side of the figure” and a suppurative granuloma “on the right side of the figure” (H&E, ×40). (b) A nonnecrotizing granuloma composed of epithelioid histiocytes, few multinucleated giant cells, and a thin rim of lymphocytes (H&E, ×100). (c) Another epithelioid granuloma with central suppurative inflammation composed of numerous neutrophils (H&E, ×100).

The histopathological examination of the right ovarian mass revealed infiltration of the ovarian stroma by a mixture of inflammatory cells composed of lymphocytes, plasma cells, neutrophils, and histiocytes, with formation of multiple microabscesses. Collections of epithelioid histiocytes with formation of granulomas were also noted. Some of these granulomas showed central suppuration. There was no evidence of malignancy. Ziehl-Neelsen and Grocott's methenamine silver stains were performed and found to be negative for acid-fast *Bacilli* and fungal organisms. The final pathological impression was of a diffuse multifocal necrotizing granulomatous inflammation that was consistent with CD (see Figure 1).

## 3. Discussion and Review of the Literature

There are 18 reported cases of ovarian CD in the literature. Gawron et al. reported a case in 2017 that described a 27-year-old nulligravida patient that was known to have CD who presented with fever, pelvic pain, and a palpable mass. Transabdominal ultrasound confirmed this mass to be located in the right iliac fossa, and hence, a tubo-ovarian abscess was suspected. As a result, the patient underwent diagnostic laparoscopy; a right-sided oophorectomy was performed and upon examining the surgical sample, a granuloma was identified and was suggestive of CD [12].

In August 2016, a total of three cases of ovarian CD were also reported, there were pelvic abscesses documented in the first two cases because of fistulization with the terminal ileum, and an ovarian abscess was noticed in the third case as a consequence of disease adjacency [13]. Rindos et al. reported in 2015 on a 35-year-old nulligravida patient known to have CD who was also diagnosed with ovarian involvement; this was believed by the authors to have started from an ovario-intestinal fistula [14].

Yabushita et al. also presented a case of ileal/ovarian cystoma fistula due to CD. A 22-year-old woman was diagnosed at the age of 20 in the hospital with CD of the large and small intestine; she had right lower abdominal pain and thus was admitted for investigations; GI contrast studies showed no fistulas but CT examination suspected an intestinal ovarian cyst fistula. Surgery was performed and revealed that the ovarian cyst became a lump with the ileum and was diagnosed as ileo-ovarian fistula; partial resection of the ileum and resection of the appendix were performed [15].

Ouakaa-Kchaou et al. reported a case of a 32-year-old Caucasian female that presented with a 1-day history of lower right quadrant abdominal pain that was associated with vomiting and fever; the patient was medically and surgically free with no family history of IBD; a diagnosis of acute appendicitis was in consideration; therefore, the patient was referred to surgery; during laparotomy, a thickened segment of the terminal ileum was adherent to the right edematous enlarged ovary with direct fistulization seen between them accompanied by an ovarian abscess with an ileo-ileal fistula, which led to the suspicion of CD. This led the surgeon to perform an en bloc right salpingo-oophorectomy with a standard ileo-cecal and ileo-ileal fistula resection. On histopathology, a section of the small intestine showed patchy mucosal ulceration, submucosal fibrosis, lymphoid aggregation, fissure ulcers, and transmural inflammation, while the right ovary showed extensive necrotizing inflammation with multinucleated giant cells and epithelioid consistent and confirming CD. Tuberculosis, actinomycosis, and malignancies were excluded. The patient is reportedly doing well after 4 years of follow-up and treatment with azathioprine [16].

Andreani et al. described a case of an ovary with an intact capsule that was macroscopically abnormal in a 28-year-old female patient complaining of a 1-year history of lower abdominal pain, weight loss of 9 kg, and unformed stools. The patient was medically and surgically free and had a negative family of IBD, and her general examination and abdominal examinations were negative [17].

Monneuse et al. reported a case of a 37-year-old female patient diagnosed with CD who came complaining of lower abdominal pain on admission. A right ovarian cyst was associated with inflammatory changes of the pelvis and involved the terminal ileum and sigmoid and was detected by an abdominal gynecologic ultrasound [18].

McCluggage et al. reported four cases of ovarian granulomas that were related to CD. Three of the four cases had similar features, two of them with granulomas that had central suppuration with microabscess formation. The author described them as having fissure ulcerations, lined by inflammatory granulation tissue that extended from the ovarian surface into the parenchyma, and vegetable materials were also identified. In the fourth case that involved the left ovary, the ovarian cortex had several small, well circumscribed, and nonnecrotizing granulomas [19].

Allen and Calvert reported a case of a 58-year-old female patient presented with lower abdominal pain and diarrhea leading to a right hemicolectomy for CD; the patient was symptom-free for 23 years; during her presentation, the disease had recurred and led to the resection of 17 cm of the ileum. 5 years later, the patient had multiple short Crohn's related strictures with fistulas and abscesses into the pelvis; two areas of the ileum measuring 10 cm and 20 cm were excised; one year later, the patient presented with lower abdominal pain and subacute obstruction. The patient was unsuccessfully treated with several courses of steroids and azathioprine, leading to further investigations that showed a short area of Crohn's ileitis and two strictures. The patient underwent a stricturoplasty and a further segment of the ileum next to an inflammatory mass surrounding the ovary was excised; postoperatively, the patient did well. The resected tubo-ovarian mass weighed 48 grams measuring 5 x 5x 3 cm; it was pale with irregular areas of necrosis. Histology showed florid necrotizing and suppurative and nonsuppurative granulomatous salpingo-oophritis, due to fistulization from the adherent bowel. Multiple acute abscesses and fragments of vegetable material were found, adjacent to the fallopian tube and between these areas were many granulomas same as the ones found in Crohn's ileitis [20].

Goldberg et al. described that vesicular fistulas and salpingitis are rare complications of CD after presenting a case of oophoro-vesicular-colonic fistula secondary to CD. Interestingly, the patient reported that during menstruation, she experienced bleeding from the bladder. A single stage oophorectomy, sigmoid resection, and repair of the fistula were done; the patient had complete resolution of her symptoms and preservation of fertility [21].

Honore presented a case of a 30-year-old nulliparous woman who complained of nonspecific symptoms, diagnosed radiologically to have distal ileitis suggesting of CD; the patient had a partial ileo-colectomy along with excision of the adherent right adnexa. Suppurative oophoritis was found in relation to an ileo-ovarian fistula and a noncaseating granulomatous inflammatory response similar to what was observed in the ileum [22].

Frost et al. reported a case of a 26-year-old black female who complained of abdominal pain for 1 year; the patient was diagnosed clinically with cystic ovaries and underwent an exploratory laparotomy that revealed ileo-cecal CD with multiple adhesions to the bowel, peritoneum, and right adnexa; a biopsy of the right ovary and an appendectomy were performed. The ovary showed multiple cysts lined by granulosa cells with scattered epithelioid granulomas beneath the serosal surface [23].

Brooks and Wheeler reported a case of a 30-year-old female patient that was known to have CD who underwent resection of the bowel and adherent adnexa. Granulomatous salpingitis, oophoritis, and enterocolitis were identified histologically. A few features that were not usual for CD included the presence of an adnexal mass and florid atypical proliferation of the tubal epithelium that are similar to the changes that can be seen in tuberculous salpingitis [24].

In this case report, we present a case of ovarian CD, a rare complication that is likely a result of direct fistulization between the bowel and the ovary. As presented, similar cases were previously reported by authors from the United States, Canada, the United Kingdom, Europe, Tunisia, and Japan. Similar to many of these reported cases, our patient presented as a case of acute abdomen that was misdiagnosed as appendicitis and led to surgery. After persistence of symptoms postoperatively, the diagnosis was questioned and an exploratory laparotomy was performed and revealed an enlarged ovary. Following resection of the ovary, the histopathological examination revealed features consistent with CD.

## 4. Conclusions

Crohn's disease is a progressive disease that can result in fistulization between the bowel and other organs. Involvement of the ovary in females with Crohn's disease is a rare complication that can be missed on initial presentation. A high index of suspicion and careful examination of histological specimens are needed to accurately reach the diagnosis. Management should be individualized but surgical intervention is usually required.

## Data Availability

This data are not publicly available.
